# Return to work improves quality of life and reduces psychosocial distress after radical cystectomy: data from a contemporary series of 230 German patients

**DOI:** 10.1007/s11764-023-01387-0

**Published:** 2023-05-05

**Authors:** Henning Bahlburg, Moritz Reike, Karl Tully, Peter Bach, Marius Cristian Butea-Bocu, Florian Roghmann, Joachim Noldus, Guido Müller

**Affiliations:** 1 Department of Urology, Marien Hospital Herne, Ruhr-University Bochum, Hölkeskampring 40, 44625 Herne, Germany; 2 Center for Urological Rehabilitation, Kliniken Hartenstein, Bad Wildungen, Germany

**Keywords:** Return to work, Quality of life, Psychosocial distress, Radical cystectomy, Bladder cancer

## Abstract

**Purpose:**

This study aims to evaluate health-related quality of life (HRQoL), psychosocial distress, and return to work (RTW) 2 years after radical cystectomy (RC) and inpatient rehabilitation (IR).

**Material and methods:**

The study relied on prospectively collected data for 842 patients, who underwent 3 weeks of IR after RC and creation of an ileal conduit (IC) or ileal neobladder (INB). Validated questionnaires surveyed patients on HRQoL and psychosocial distress (EORTC QLQ-C30, QSC-R10). Furthermore, employment status was evaluated. Regression was performed to identify predictors for HRQol, psychosocial distress, and RTW.

**Results:**

Two-hundred and thirty patients were employed pre-surgery (77.8% INB, 22.2% IC). Patients with an IC suffered significantly more often from locally advanced disease (≥ pT3: 43.1% vs 22.9%; *p* = 0.004). Two years after surgery, 16.1% of patients had died (median days of survival 302 (IQR 204–482). Global HRQoL improved steadily, while high psychosocial distress was present in 46.5% of patients 2 years after surgery. Employment was reported by 68.2% of patients, of which 90.3% worked full-time. Retirement was reported by 18.5%. Multivariate logistic regression analysis identified age ≤ 59 years as the only positive predictor for RTW 2 years after surgery (OR 7.730; 95% CI 3.369–17.736; *p* < 0.001). Gender, surgical technique, tumor stage, and socioeconomic status did not influence RTW in this model. In multivariate linear regression analysis, RTW was identified as an independent predictor of better global HRQoL (*p* = 0.018) and lower psychosocial distress (*p* < 0.001), whereas younger patient age was identified as an independent predictor for higher psychosocial distress (*p* = 0.002).

**Conclusion:**

Global HRQoL and RTW are high among patients two years after RC. However, role and emotional, cognitive, and social functioning were significantly impaired, while high psychosocial distress persists in a material number of patients.

**Implications for Cancer Survivors:**

Our study highlights how a successful RTW decreases psychosocial distress and increases QoL in patients after RC for urothelial cancer. Nonetheless, additional efforts by employers and healthcare providers are needed in aftercare after creation of an INB or IC.

## Introduction

Many patients are still of working age when diagnosed with cancer. For patients with organ-confined muscle-invasive or high-risk non-muscle invasive bladder cancer, radical cystectomy (RC) represents an established therapeutic option [1, 2]. In Germany, RC increased by 28% between 2006 and 2019 [3]. Five-year survival after RC is between 25 and 72%, depending on tumor stage [4]. For urinary diversion, ileal conduit (IC) or ileal neobladder (INB) is well established [5–7]. Employment is linked to higher health-related quality of life (HRQoL) in cancer patients, as a return to work (RTW) after therapy signals successful disease management as well as reintegration into family and social networks [8–10]. Diminished QoL itself can influence survival [11, 12]. Dependent on tumor type, up to 60% of patients suffer from high depression, anxiety, or an adjustment disorder [13]. Accordingly, psychosocial distress has been discussed as a “sixth vital sign” in cancer treatment [14]. Guidelines recommend psycho-oncological support and counseling as standard components of cancer treatment [15].

To enable reintegration into daily life and employment, German social laws entitle cancer patients to an average of 3 weeks of inpatient rehabilitation (IR). The Guideline of the German Society of Urology recommends that all patients be offered several weeks of IR after RC for bladder cancer to minimize functional disorders, reduce psychological stress, and improve HRQoL [2]. It is assumed that in Germany almost all patients participate in IR as recommended. However, data in this regard is lacking. The need for rehabilitation after RC is supported by recently published data, which reveals significantly higher rates of postoperative complications than previously reported in the literature. In addition, a high percentage of complications occur after discharge from the primary hospital [16]. This prospective study aims to investigate RTW, HRQoL, and psychosocial distress in a contemporary series of patients after RC and creation of an IC or an INB.

## Methods

This prospective study is based on clinical data of patients with urothelial carcinoma of the bladder who underwent RC and IC or INB creation in various hospitals across Germany, followed by IR in a specialized center for urological rehabilitation (Kliniken Hartenstein, Bad Wildungen, Germany) between April 2018 and December 2019. The study protocol was approved by an institutional research committee (research authorization number FF30/2017). Validated questionnaires surveyed patients on HRQoL and psychosocial distress until 2 years after RC. Furthermore, employment status was evaluated.

Baseline characteristics comprised patients’ age, Karnofsky performance status, body mass index (BMI), the existence of cardiovascular disease and/or diabetes, tumor stage, method of surgery, utilization of neoadjuvant chemotherapy, and socioeconomic status [17].

### Inpatient rehabilitation (IR)

During IR, psychosocial interventions were performed by psychologists and social worker in addition to physiotherapy and urologic counseling. The program includes information on bladder cancer and aftercare, individual, group, and couple psychotherapy, relaxation training, and psychoeducation.

### Quality of Life Questionnaire (EORTC QLQ-C30)

The EORTC QLQ-C30, issued by the European Organization for Research and Treatment of Cancer (EORTC), is a questionnaire designed to evaluate QoL in cancer patients [18]. A high score in functional scales mirrors high quality of life. Normative data on the QoL of the general German population were used for comparison [19].

### Questionnaire on Stress in Cancer Patients (QSC-R10)

Patients were screened for psychosocial distress using the Questionnaire on Stress in Cancer Patients (QSC-R10), a standardized and validated 10-item self-assessment instrument [20]. The 10 items cover the most relevant psychosocial aspects of everyday life in cancer patients and are answered on a scale of 0 (“not applicable”) to 5 (“very high burden”). The QSC-R10 total score is calculated by adding up the single items. A sum ≥ 15 mirrors a high psychosocial distress.

### Statistical analysis

Descriptive analyses were performed to examine sample characteristics and to describe RTW outcomes. Between-group comparisons were analyzed using the Mann–Whitney *U* test or Chi-square test (Pearson) as appropriate. Log-rank test was employed to analyze survival. Multivariate regression analyses were performed to identify predictors for HRQoL, psychosocial distress, and RTW. Significance was considered at *p* < 0.05. Analyses were performed by using IBM SPSS version 29.

## Results

A total of 842 patients in the overall cohort underwent RC in 135 different hospitals in Germany. Two hundred and thirty patients (27.3%) were employed before surgery. Their characteristics are summarized in Table 1.

**Table 1:** Baseline characteristics of 230 employed patients after radical cystectomy

Variable	Total	Conduit	Neobladder	p*
Patients, *n* (%)	230 (100.0)	51 (22.2)	179 (77.8)	
Age (years), median (IQR)	58 (55–62)	61 (57–62)	58 (55–61)	0.099
Gender, *n* (%)				
Male	191 (83.0)	27 (52.9)	164 (91.6)	** < 0.001**
Female	39 (17.0)	24 (47.1)	15 (8.4)	** < 0.001**
Karnofsky performance status (%), median (IQR)	80 (70–80)	80 (70–80)	80 (70–80)	0.508
BMI (kg/m^2^), median (IQR)	25 (23–28)	25 (21–29)	25 (23–27)	0.741
≥ 30, *n* (%)	26 (11.3)	9 (17.6)	17 (9.5)	0.105
Cardiovascular disease, *n* (%)	97 (42.2)	22 (43.1)	75 (41.9)	0.875
Diabetes, *n* (%)	15 (6.5)	1 (2.0)	14 (7.8)	0.135
Socioeconomic status, *n* (%)**				
Low	101 (45.3)	31 (64.6)	70 (40.0)	**0.002**
Middle	95 (42.6)	16 (33.3)	79 (45.1)	0.143
High	27 (12.1)	1 (2.1)	26 (14.9)	**0.016**
Neoadjuvant chemotherapy, *n* (%)	36 (15.7)	9 (17.6)	27 (15.1)	0.657
Method of surgery, *n* (%)				
Robot-assisted cystectomy	27 (11.7)	6 (11.8)	21 (11.7)	0.995
Open cystectomy	203 (88.3)	45 (88.2)	158 (88.3)	0.995
Tumor stage, *n* (%)				
≥ pT3a	63 (27.4)	22 (43.1)	41 (22.9)	**0.004**
Lymph node positive, *n* (%)***	35 (15.9)	12 (23.5)	23 (13.6)	0.090
No. of lymph nodes removed, median (IQR)	17 (11–24)	16 (12–24)	18 (11–24)	0.762

All entries with boldface in the tables show significant results (*p* < 0.05)

Abbreviations: *IQR*, interquartile range. *BMI*, body mass index

^*^Mann–Whitney *U* test or Chi-square test (Pearson) as appropriate

^**^Data available for 223 patients (conduit *n* = 48 and neobladder *n* = 175)

^***^Data available for 220 patients (conduit *n* = 51 and neobladder *n* = 169)

Median age was 58 years (IQR 55–61) in patients with an INB and 61 years (IQR 57–62) in patients with an IC (*p* = 0.099). INB was chosen for 77.8% of employed patients, while 22.2% received an IC. Men received an INB significantly more often as urinary diversion (85.9% vs 14.1%, *p* < 0.001). Conversely, women received an IC more often (61.5% vs 38.5%, *p* < 0.001). Locally advanced disease (≥ pT3) was found significantly more often in patients with an IC (43.1% vs 22.9%; *p* = 0.004). Lymph node metastases were found in 23.5% of IC patients and 13.6% of patients with an INB—however, without a significance level (*p* = 0.09). Patients with low socioeconomic status were far more likely to receive an IC (64.6% vs 40.0%; *p* = 0.002), as patients with high socioeconomic status received an INB significantly more often (14.9% vs 2.1%, *p* = 0.016). There were no group differences between the two types of urinary diversion concerning Karnofsky performance status (median 80%), BMI (median 25 kg/m^2^), and the proportion of cardiovascular disease (42.2%), diabetes mellitus (6.5%), neoadjuvant chemotherapy (15.7%), or robotic surgery (11.7%).

**Fig. 1:**
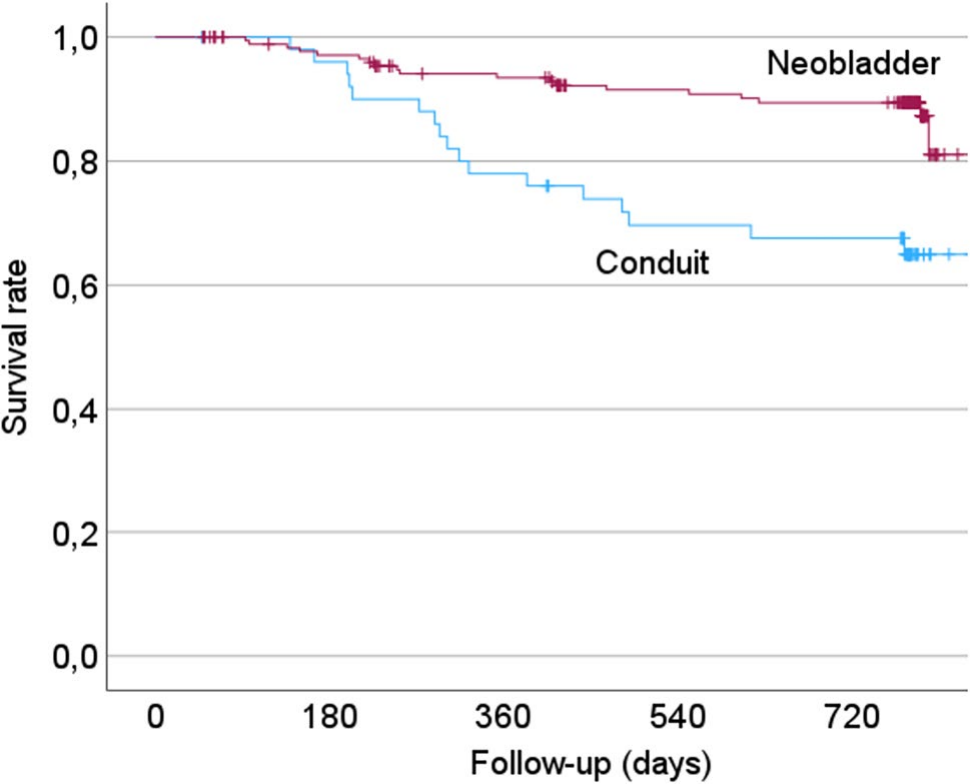
Survival rates of employed patients after radical cystectomy

During follow-up, 37 of previously employed patients (16.1%) died. Log-rank test revealed a significantly higher probability of survival in patients with an INB (Chi-square = 14.65; *p* < 0.001; Fig. 1). Response rate for the follow-up survey 2 years after surgery was 69.1%. Eight patients did not report their employment status 2 years after RC.

### Quality of life

**Fig. 2:**
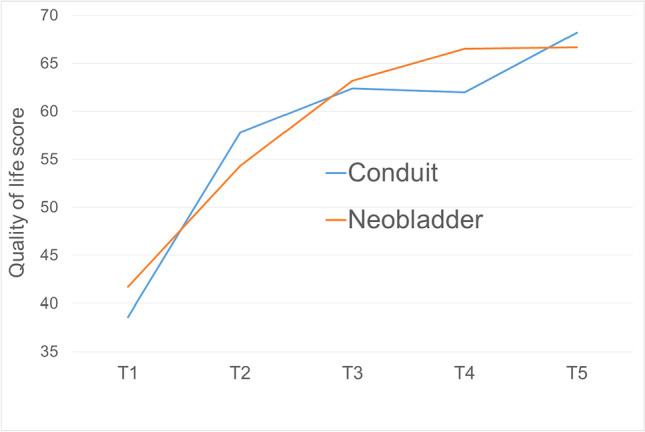
Health-related quality of life (EORTC QLQ-C30) in preoperatively employed patients after radical cystectomy. Abbreviations: T1 = beginning of inpatient rehabilitation (median 28 days (IQR 23–35) after surgery); *n* = 230; T2 = end of inpatient rehabilitation (median 54 days (IQR 48–62) after surgery); *n* = 230; T3 = 6 months after surgery; *n* = 188; T4 = 1 year after surgery; *n* = 156; T5 = 2 years after surgery; *n* = 159

Global HRQoL improved continuously and significantly up to two years after surgery (Fig. 2).

No significant differences were detected between the two types of urinary diversion. Data on HRQol and QoL functional scales in comparison to normative data can be found in Table 2.

**Table 2:** QLQ-C30 functional scales 2 years after surgery in patients who were employed before radical cystectomy: comparison with normative data (60–69 years)

Variable	Total mean (SD)	Conduit mean (SD)	Neobladder mean (SD)	Normative data (mean)	
				Male	Female
Global health status/quality of life	67.0 (21.3)	68.2 (20.8)	66.7 (21.4)	65.9	65.5
*p* = 0.801					
Physical functioning	80.2 (20.0)	76.7 (19.8)	81.0 (20.0)	83.0	81.0
*p* = 0.151					
Role functioning	62.2 (29.3)	58.6 (29.5)	63.1 (29.4)	78.6	77.3
*p* = 0.441					
Emotional functioning	64.9 (27.3)	61.6 (29.9)	65.7 (26.6)	75.7	72.8
*p* = 0.491					
Cognitive functioning	75.9 (25.2)	78.5 (27.3)	75.3 (24.7)	85.1	87.1
*p* = 0.340					
Social functioning	60.9 (30.1)	60.2 (30.0)	61.1 (30.2)	80.6	83.1
*p* = 0.795					

Abbreviation: *SD*, standard deviation

Two years after surgery, global HRQoL (mean 67.0) and physical functioning (mean 80.2) did not differ from the general German population. However, there was a moderate to severe impairment in emotional (mean 64.9), role (mean 62.2), cognitive (mean 75.9), and social functioning (mean 60.9), respectively. Again, no significant differences were detected between the two types of urinary diversion.

In multivariate linear regression analysis, RTW was identified as an independent predictor for better global HRQoL 2 years after surgery (Table 3). The mean global HRQoL is expected to be about 10 points higher in patients with RTW than in patients without RTW (*p* = 0.018). Urinary diversion, sex, age, tumor stage, or lymph node metastases were not identified as predictors in this model.

**Table 3:** Regression analyses to identify independent predictors of (a) global HRQoL, (b) psychosocial distress, and (c) RTW 2 years after radical cystectomy and urinary diversion

(a) Global HRQoL	*t*	Regression coefficient	95% CI	*p*
Neobladder	− 0.825	− 3.923	− 13.328 to 5.483	0.411
Male	− 0.144	− 0.830	− 12.227 to 10.566	0.886
Age	1.070	0.424	− 0.359 to 1.207	0.287
Tumor stage ≥ pT3	− 0.269	− 1.260	− 10.521 to 8.001	0.788
Positive nodal stage	− 0.893	− 5.262	− 16.916 to 6.393	0.374
RTW	2.403	10.266	1.820 to 18.712	**0.018**
(b) Psychosocial distress	*t*	Regression coefficient	95% CI	*p*
Neobladder	− 0.397	− 0.922	− 5.518 to 3.674	0.692
Male	0.835	2.356	− 3.223 to 7.934	0.405
Age	− 3.200	− 0.610	− 0.987 to − 0.233	**0.002**
Tumor stage ≥ pT3	0.783	1.767	− 2.694 to 6.227	0.435
Positive nodal stage	0.765	2.200	− 3.488 to 7.888	0.446
RTW	− 3.266	− 6.702	− 10.759 to − 2.645	**0.001**
(c) Return to work	Univariate		Multivariate	
	OR (95% CI)	*p*	OR (95% CI)	*p*
Age ≤ 59 years (yes vs. no)	7.212 (3.337–15.583)	** < 0.001**	7.730 (3.369–17.736)	** < 0.001**
Male vs. female	1.182 (0.439–3.182)	0.741	0.920 (0.247–3.427)	0.901
Neobladder vs. conduit	2.416 (1.054–5.534)	**0.037**	1.578 (0.541–4.605)	0.403
Robotic vs. open cystectomy	1.134 (0.376–3.422)	0.823	0.693 (0.194–2.483)	0.574
Tumor stage ≤ pT2 (yes/no)	1.504 (0.642–3.521)	0.347	1.464 (0.500–4.286)	0.487
Positive nodal stage (yes/no)	0.398 (0.135–1.172)	0.095	0.454 (0.121–1.696)	0.240
High socioeconomic status (yes/no)	2.933 (0.815–10.552)	0.099	3.735 (0.906–15.407)	0.068

All entries with boldface in the tables show significant results (*p *< 0.05)

Abbreviations: *HRQoL*, health-related quality of life. *RTW*, return to work. *OR*, odds ratio. *CI*, confidence interval

### Psychosocial distress

**Fig. 3:**
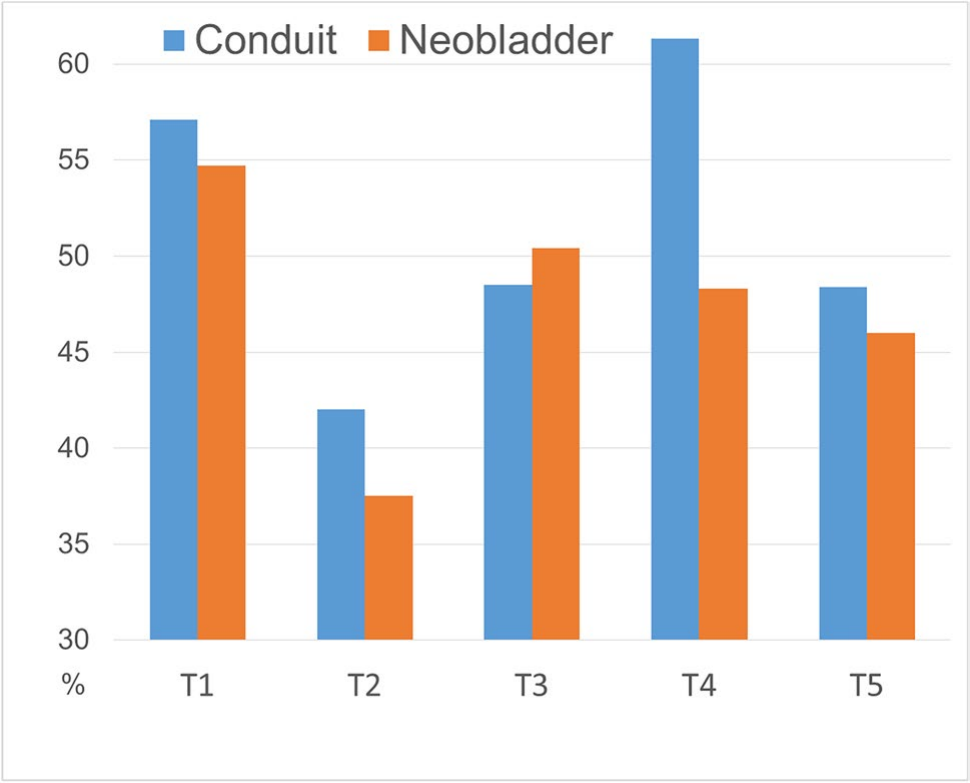
High psychosocial distress (QSC-R10 score ≥ 15) in preoperatively employed patients after radical cystectomy. Abbreviations: T1 = beginning of inpatient rehabilitation (median 28 days (IQR 23–35) after surgery); *n* = 230; T2 = end of inpatient rehabilitation (median 54 days (IQR 48–62) after surgery); *n* = 230; T3 = 6 months after surgery; *n* = 188; T4 = 1 year after surgery; *n* = 156; T5 = 2 years after surgery; *n* = 159

Initially, the percentage of patients with high psychosocial distress decreased significantly in all patients during IR from 55.3 to 38.5% (*p* < 0.001), only to increase again during further follow-up. Six months after surgery, 50.0% of employed patients suffered from high psychosocial distress. Levels of distress remained elevated during further follow-up. One year after RC, 51% of patients, and two years after RC, 46.5% of patients still suffered from high psychosocial distress. Psychosocial distress did not differ significantly between the two types of urinary diversion at any time during the evaluation (Fig. 3).

Multivariate linear regression analysis identified age (*p* = 0.002) and RTW (*p* < 0.001) as independent predictors of psychosocial distress 2 years after RC (Table 3). The QSC-R10 score is expected to be 7 points lower in patients with RTW than in patients without RTW, while the score is expected to decrease by 0.610 points with every year of life. Urinary diversion, sex, tumor stage, or lymph node metastases appear to not affect psychosocial distress in this model.

### Return to work

Two years after surgery, 68.2% (*n* = 103) of patients employed pre-surgery were re-integrated into work successfully, while 18.5% (*n* = 28) retired. Of those employed, 90.3% (*n* = 93) worked full-time, while 9.7% (*n* = 10) worked part-time. Fourteen patients (9.3%) were unemployed 2 years after surgery. Disability pension was received by 4% (*n* = 6).

Univariate logistic regression analysis identified age ≤ 59 years (OR 7.212; 95% CI 3.337–15.583; *p* < 0.001) and INB (OR 2.416; 95% CI 1.054–5.434; *p* = 0.037) as positive predictors for RTW, while multivariate logistic regression analysis identified age ≤ 59 years as the only positive predictor for RTW 2 years after RC (OR 7.730; 95% CI 3.369–17.736; *p* < 0.001). High socioeconomic status trended towards significance (*p* = 0.068). Sex, surgical technique, tumor stage, and lymph node metastases did not influence RTW (Table 3).

## Discussion

Taking age-adjusted population-based normative data into account, HRQoL and physical functioning of patients in this study were comparable to the general German population 2 years after RC and urinary diversion [19]. However, moderate to high impairment of emotional, role, social, and cognitive functioning was observed [21]. Meanwhile, a “response shift” with subsequent adaption may explain why impaired symptom scales do not negatively influence QoL [22]. The mean scores of global HRQoL and functional QoL scales did not differ significantly between patients with an IC and INB, respectively. Abozaid et al. state that physical functioning can take up to 12 months to recover after RC [23]. In our study, physical functioning 1 year after surgery is comparable to normative data from the general German population and therefore corroborates this result. Venkatramani et al. showed that there was no difference in the recovery of activities of daily life when comparing patients after robot-assisted RC with patients after conventional RC [24]. As the focus is commonly on global HRQol and physical functioning, additional efforts to also restore role, social, emotional, and cognitive functioning are required by healthcare providers. As generic questionnaires may not detect cancer-specific issues sufficiently, an analysis of QoL in cancer patients should always consider certain subscales that may not affect the healthy percentage of the population.

In our study, RTW was identified as the only independent predictor for better HRQoL 2 years after RC. A higher QoL in cancer survivors re-integrated into the workforce is backed up by the aforementioned publications by Kennedy et al., de Boer et al., and Hoffmann et al. [8–10].

Since financial and job insecurities are known to influence QoL and may lead to depression, a successful RTW should be aspired for cancer survivors [25]. Two years after surgery, a successful RTW was documented in 68.2% of patients in our study, with 90.3% working full-time. Univariate regression analysis identified age ≤ 59 years (OR 7.212; 95% CI 3.337–15.583; *p* < 0.001) and INB (OR 2.416; 95% CI 1.054–5.434; *p* = 0.037) as positive predictors for RTW, while multivariate regression analysis identified age ≤ 59 years as the only positive predictor for RTW (OR 7.730; 95% CI 3.369–17.736; *p* < 0.001). A significant difference between white-and blue-collar workers in terms of RTW rates is reported in the literature. Lower education, manual work, and lower income decrease the probability of RTW [26–28]. In our model, high socioeconomic status showed only a trend towards higher RTW rates (*p* = 0.068). As the current RTW rate in German cancer patients is 61–64%, a RTW in 68.2% of patients included in this study is quite acceptable [29, 30]. But RTW in German cancer patients is notoriously lower than in countries such as the Netherlands, France, the UK, or the USA [27, 31]. This may be explained by the various social services offered to patients with consequential damage through illness [32]. However, (neo-) adjuvant chemotherapy with its associated adverse long-term effects such as peripheral neuropathy, rapid fatigability, cognitive impairment, and motor impairment may further obstruct a successful RTW [33–35]. Our collective had a mean age of 61 years (IC) and 58 years (INB) at the time of surgery, so a retirement rate of 18.5% 2 years after surgery is not surprising. The current rate of unemployment in Germany among citizens between 55 and 65 years of age is 6.1% [36]. It is known that cancer survivors have a significantly higher risk of unemployment [32]. Therefore, the unemployment rate of 9.7% 2 years after RC in our study should not go unnoticed. Among current 55 year olds, 47.3% of men and 38.8% of women are expected to develop cancer within the next 10 years. However, survival rates have increased significantly [37]. Improved efforts from both employers and governing bodies are needed to enable RTW since more survivors will need to be re-integrated into the workforce [38]. Low RTW rates may cause diminished QoL and increased psychosocial distress, resulting in further strains on healthcare providers and social funds. IR plays an important role in restoring physical and mental function in cancer patients and lays the foundation for a successful RTW. Due to saved disability pensions and collected taxes and contributions, the cost of rehabilitation measures will amortize after 4 months [32].

Two years after RC, 46.5% of patients in our study suffered from high psychosocial distress. RTW was identified as an independent predictor of lower psychosocial distress, whereas younger patient age was identified as an independent predictor for higher psychosocial distress. Up to 89% of cancer patients lament insufficient psychosocial care [39]. Thus, psychosocial and psycho-oncological counseling should be made easily accessible and affordable. Healthcare providers should openly talk about and refer patients to counseling sessions and self-help groups if the need arises. Special focus should be given to patients that were not successfully re-integrated into the workforce. The German healthcare system offers the option of a renewed oncological rehabilitation measure if deemed necessary by treating physicians and patients.

Naturally, there are some limitations to our study. As IR is specific to the German healthcare system, a generalization of results from this cohort to patients undergoing treatment in different healthcare systems should be conducted cautiously. Furthermore, HRQoL and psychosocial distress were not measured pre-surgery. Palapattu et al. reported that up to 45% of patients undergoing RC suffer from high psychosocial distress in the perioperative period [40]. During follow-up, 42 patients received adjuvant or palliative chemotherapy. However, the impact of (neo-) adjuvant or palliative chemotherapy on HRQoL, psychosocial distress, and RTW was not assessed in this study. Furthermore, data on gradual reintegration, which has been identified as a strong predictor for RTW, is lacking in this study [41]. Data on type and frequency of psychosocial aftercare after discharge from IR were not recorded in our study, and there was no control group outside of IR. Nonetheless, our data report HRQoL, psychosocial distress, and RTW rates in a large number of patients after RC with IC or INB creation in a short and recent period, and in a multi-institutional approach. Our study highlights the need for sufficient psychosocial counseling and aftercare during all stages of disease as well as the importance of a successful RTW.

## Conclusion

While global HRQoL and physical functioning in patients 2 years after RC and urinary diversion for IC or INB are comparable to the general German population, emotional, role, cognitive, and social functioning are still significantly impaired. The RTW rate is high compared to other malignancies. Nonetheless, additional efforts by both employers and regulators are needed to successfully re-integrate more and especially older cancer survivors into the workforce. Furthermore, as psychosocial distress remains high in a substantial number of patients, both psychosocial counseling and participation in self-help groups should be encouraged.

## Data Availability

The data that support the findings of this study are not publicly available but may be made available on request from the corresponding author, HB.

## References

[1] Chang SS, Bochner BH, Chou R, Dreicer R, Kamat AM, Lerner SP, et al. Treatment of non-metastatic muscle-invasive bladder cancer: AUA/ASCO/ASTRO/SUO Guideline. J Urol. 2017;198:552–9. 28456635 10.1016/j.juro.2017.04.086 PMC5626446

[2] Leitlinienprogramm Onkologie (Deutsche Krebsgesellschaft, Deutsche Krebshilfe, AWMF): S3-Leitlinie Früherkennung, Diagnose, Therapie und Nachsorge des Harnblasenkarzinoms, Langversion 2.0, AWMF-Registrierungsnummer 032/038OL. 2020. https://www.leitlinienprogramm-onkologie.de/leitlinien/harnblasenkarzinom/. abgerufen am: 02.03.2023.

[3] Flegar L, Kraywinkel K, Zacharis A, Aksoy C, Koch R, Eisenmenger N, et al. Treatment trends for muscle-invasive bladder cancer in Germany from 2006 to 2019. World J Urol. 2022;40:1715–21. 35486177 10.1007/s00345-022-04017-z PMC9237006

[4] Ploussard G, Shariat SF, Dragomir A, Kluth LA, Xylinas E, Masson-Lecomte A, et al. Conditional survival after radical cystectomy for bladder cancer: evidence for a patient changing risk profile over time. Eur Urol. 2014;66:361–70. 24139235 10.1016/j.eururo.2013.09.050

[5] Frohneberg D, Bachor R, Egghart G, Miller K, Hautmann R. Ileal neobladder. Principles of function and continence. Eur Urol. 1989;16:241–9. 2767091 10.1159/000471584

[6] Hautmann RE, Abol-Enein H, Davidsson T, Gudjonsson S, Hautmann SH, Holm HV, et al. ICUD-EAU International Consultation on Bladder Cancer 2012: urinary diversion. Eur Urol. 2013;63:67–80. 22995974 10.1016/j.eururo.2012.08.050

[7] Todenhofer T, Stenzl A, Schwentner C. Optimal use and outcomes of orthotopic neobladder reconstruction in men and women. Curr Opin Urol. 2013;23:479–86. 23851385 10.1097/MOU.0b013e328363f6e9

[8] Kennedy F, Haslam C, Munir F, Pryce J. Returning to work following cancer: a qualitative exploratory study into the experience of returning to work following cancer. Eur J Cancer Care (Engl). 2007;16:17–25. 17227349 10.1111/j.1365-2354.2007.00729.x

[9] de Boer AG, Verbeek JH, Spelten ER, Uitterhoeve AL, Ansink AC, de Reijke TM, et al. Work ability and return-to-work in cancer patients. Br J Cancer. 2008;98:1342–7. 18349834 10.1038/sj.bjc.6604302 PMC2361697

[10] Hoffman B. Cancer survivors at work: a generation of progress. CA Cancer J Clin. 2005;55:271–80. 16166073 10.3322/canjclin.55.5.271

[11] Coates A, Porzsolt F, Osoba D. Quality of life in oncology practice: prognostic value of EORTC QLQ-C30 scores in patients with advanced malignancy. Eur J Cancer. 1997;33:1025–30. 9376182 10.1016/S0959-8049(97)00049-X

[12] Roychowdhury DF, Hayden A, Liepa AM. Health-related quality-of-life parameters as independent prognostic factors in advanced or metastatic bladder cancer. J Clin Oncol. 2003;21:673–8. 12586805 10.1200/JCO.2003.04.166

[13] Peters L, Brederecke J, Franzke A, de Zwaan M, Zimmermann T. Psychological distress in a sample of inpatients with mixed cancer-a cross-sectional study of routine clinical data. Front Psychol. 2020;11:591771. 33329254 10.3389/fpsyg.2020.591771 PMC7734182

[14] Bultz BD, Carlson LE. Emotional distress: the sixth vital sign in cancer care. J Clin Oncol. 2005;23:6440–1. 16155033 10.1200/JCO.2005.02.3259

[15] Leitlinienprogramm Onkologie (Deutsche Krebsgesellschaft DK, AWMF). Psychoonkologische Diagnostik, Beratung und Behandlung von erwachsenen Krebspatient*innen. Langversion 2.01 (Konsultationsfassung), 2022, AWMF- Registernummer: 032/051OL https://www.leitlinienprogramm-onkologie.de/leitlinien/psychoonkologie/; Zugriff am 19.02.2023. 2014.

[16] Gotte M, Bahlburg H, Butea-Bocu MC, von Landenberg N, Tully K, Roghmann F et al. Complications in the early recovery period after radical cystectomy-real data from impartial inpatient rehabilitation. Clin Genitourin Cancer. 2022;20:e424–e31. 10.1016/j.clgc.2022.05.008 35691884

[17] Winkler JSH. Der Sozialschicht-Index im Bundes-Gesundheitssurvey. Gesundheitswesen. 1999;61:178–83. 10726418

[18] Aaronson NK, Ahmedzai S, Bergman B, Bullinger M, Cull A, Duez NJ, et al. The European Organization for Research and Treatment of Cancer QLQ-C30: a quality-of-life instrument for use in international clinical trials in oncology. J Natl Cancer Inst. 1993;85:365–76. 8433390 10.1093/jnci/85.5.365

[19] Waldmann A, Schubert D, Katalinic A. Normative data of the EORTC QLQ-C30 for the German population: a population-based survey. PLoS One. 2013;8:e74149. 24058523 10.1371/journal.pone.0074149 PMC3769241

[20] Book K, Marten-Mittag B, Henrich G, Dinkel A, Scheddel P, Sehlen S, et al. Distress screening in oncology-evaluation of the Questionnaire on Distress in Cancer Patients-short form (QSC-R10) in a German sample. Psychooncology. 2011;20:287–93. 20669340 10.1002/pon.1821

[21] Osoba D, Rodrigues G, Myles J, Zee B, Pater J. Interpreting the significance of changes in health-related quality-of-life scores. J Clin Oncol. 1998;16:139–44. 9440735 10.1200/JCO.1998.16.1.139

[22] Schwartz CE, Bode R, Repucci N, Becker J, Sprangers MA, Fayers PM. The clinical significance of adaptation to changing health: a meta-analysis of response shift. Qual Life Res. 2006;15:1533–50. 17031503 10.1007/s11136-006-0025-9

[23] Abozaid M, Tan WS, Khetrapal P, Baker H, Duncan J, Sridhar A, et al. Recovery of health-related quality of life in patients undergoing robot-assisted radical cystectomy with intracorporeal diversion. BJU Int. 2022;129:72–9. 34092021 10.1111/bju.15505

[24] Venkatramani V, Reis IM, Gonzalgo ML, Castle EP, Woods ME, Svatek RS, et al. Comparison of robot-assisted and open radical cystectomy in recovery of patient-reported and performance-related measures of independence: a secondary analysis of a randomized clinical trial. JAMA Netw Open. 2022;5:e2148329. 35171260 10.1001/jamanetworkopen.2021.48329 PMC8851298

[25] Catto JWF, Downing A, Mason S, Wright P, Absolom K, Bottomley S, et al. Quality of life after bladder cancer: a cross-sectional survey of patient-reported outcomes. Eur Urol. 2021;79:621–32. 33581875 10.1016/j.eururo.2021.01.032 PMC8082273

[26] van Muijen P, Weevers NL, Snels IA, Duijts SF, Bruinvels DJ, Schellart AJ, et al. Predictors of return to work and employment in cancer survivors: a systematic review. Eur J Cancer Care (Engl). 2013;22:144–60. 23279195 10.1111/ecc.12033

[27] Mehnert A, Koch U. Predictors of employment among cancer survivors after medical rehabilitation–a prospective study. Scand J Work Environ Health. 2013;39:76–87. 22422040 10.5271/sjweh.3291

[28] Taskila-Brandt T, Martikainen R, Virtanen SV, Pukkala E, Hietanen P, Lindbohm ML. The impact of education and occupation on the employment status of cancer survivors. Eur J Cancer. 2004;40:2488–93. 15519524 10.1016/j.ejca.2004.06.031

[29] Mehnert A. Employment and work-related issues in cancer survivors. Crit Rev Oncol Hematol. 2011;77:109–30. 20117019 10.1016/j.critrevonc.2010.01.004

[30] Funke A ES, Streibelt M, Steinbrecher A. Deutsche Rentenversicherung Bund (Reha-Bericht 2021): Die medizinische und berufliche Rehabilitation der Rentenversicherung im Licht der Statistik. 2021.

[31] Short PF, Vasey JJ, Tunceli K. Employment pathways in a large cohort of adult cancer survivors. Cancer. 2005;103:1292–301. 15700265 10.1002/cncr.20912

[32] Rick O, Kalusche EM, Dauelsberg T, Konig V, Korsukewitz C, Seifart U. Reintegrating cancer patients into the workplace. Dtsch Arztebl Int. 2012;109:702–8. 23264814 10.3238/arztebl.2012.0702 PMC3489075

[33] Go RS, Adjei AA. Review of the comparative pharmacology and clinical activity of cisplatin and carboplatin. J Clin Oncol. 1999;17:409–22. 10458260 10.1200/JCO.1999.17.1.409

[34] Ahles TA, Root JC, Ryan EL. Cancer- and cancer treatment-associated cognitive change: an update on the state of the science. J Clin Oncol. 2012;30:3675–86. 23008308 10.1200/JCO.2012.43.0116 PMC3675678

[35] Wefel JS, Schagen SB. Chemotherapy-related cognitive dysfunction. Curr Neurol Neurosci Rep. 2012;12:267–75. 22453825 10.1007/s11910-012-0264-9

[36] Statistik der Bundesagentur für Arbeit Berichte: Blickpunkt Arbeitsmarkt – Situation Älterer am Arbeitsmarkt, Nürnberg. 2022.

[37] Barnes B KK, Nowossadeck E, Schönfeld I, Starker A, Wienecke A, Wolf U. Bericht zum Krebsgeschehen in Deutschland 2016. Bericht zum Krebsgeschehen in Deutschland 2016. 2016. 10.17886/rkipubl-2016-014

[38] Taskila T, Lindbohm ML, Martikainen R, Lehto US, Hakanen J, Hietanen P. Cancer survivors’ received and needed social support from their work place and the occupational health services. Support Care Cancer. 2006;14:427–35. 16402234 10.1007/s00520-005-0005-6

[39] Harrison JD, Young JM, Price MA, Butow PN, Solomon MJ. What are the unmet supportive care needs of people with cancer? A systematic review. Support Care Cancer. 2009;17:1117–28. 19319577 10.1007/s00520-009-0615-5

[40] Palapattu GS, Haisfield-Wolfe ME, Walker JM, BrintzenhofeSzoc K, Trock B, Zabora J, et al. Assessment of perioperative psychological distress in patients undergoing radical cystectomy for bladder cancer. J Urol. 2004;172:1814–7. 15540727 10.1097/01.ju.0000141245.08456.1a

[41] Rick O. Importance of gradual reintegration for return to work in oncology patients. Rehabilitation (Stuttg). 2022;61:117–24. 34592774 10.1055/a-1578-1449

